# Unveiling the Phytochemical Profile and Biological Potential of Five *Artemisia* Species

**DOI:** 10.3390/antiox11051017

**Published:** 2022-05-21

**Authors:** Adriana Trifan, Gokhan Zengin, Kouadio Ibrahime Sinan, Elwira Sieniawska, Rafal Sawicki, Magdalena Maciejewska-Turska, Krystyna Skalikca-Woźniak, Simon Vlad Luca

**Affiliations:** 1 Department of Pharmacognosy, *Grigore T. Popa* University of Medicine and Pharmacy Iasi, 700115 Iasi, Romania; adriana.trifan@umfiasi.ro; 2 Physiology and Biochemistry Research Laboratory, Department of Biology, Science Faculty, Selcuk University, University Campus, 42130 Konya, Turkey; gokhanzengin@selcuk.edu.tr (G.Z.); sinankouadio@gmail.com (K.I.S.); 3 Department of Natural Products Chemistry, Medical University of Lublin, 20-093 Lublin, Poland; kskalicka@pharmacognosy.org; 4 Department of Biochemistry and Biotechnology, Medical University of Lublin, 20-093 Lublin, Poland; rafal.sawicki@umlub.pl; 5 Department of Pharmacognosy with the Medicinal Plant Garden, Medical University of Lublin, 20-093 Lublin, Poland; magdalena.maciejewska@umlub.pl; 6 Biothermodynamics, TUM School of Life Sciences, Technical University of Munich, 85354 Freising, Germany

**Keywords:** *Artemisia*, LC-HRMS/MS, chlorogenic acids, artemisinin, enzyme inhibitory, *Mycobacterium*, multivariate analysis

## Abstract

The *Artemisia* L. genus comprises over 500 species with important medicinal and economic attributes. Our study aimed at providing a comprehensive metabolite profiling and bioactivity assessment of five *Artemisia* species collected from northeastern Romania (*A. absinthium* L., *A. annua* L., *A. austriaca* Jacq., *A. pontica* L. and *A. vulgaris* L.). Liquid chromatography–tandem high-resolution mass spectrometry (LC-HRMS/MS) analysis of methanol and chloroform extracts obtained from the roots and aerial parts of the plants led to the identification of 15 phenolic acids (mostly hydroxycinnamic acid derivatives), 26 flavonoids (poly-hydroxylated/poly-methoxylated flavone derivatives, present only in the aerial parts), 14 sesquiterpene lactones, 3 coumarins, 1 lignan and 7 fatty acids. Clustered image map (CIM) analysis of the phytochemical profiles revealed that *A. annua* was similar to *A. absinthium* and that *A. pontica* was similar to *A. austriaca,* whereas *A. vulgaris* represented a cluster of its own. Correlated with their total phenolic contents, the methanol extracts from both parts of the plants showed the highest antioxidant effects, as assessed by the DPPH and ABTS radical scavenging, CUPRAC, FRAP and total antioxidant capacity methods. *Artemisia* extracts proved to be promising sources of enzyme inhibitory agents, with the methanol aerial part extracts being the most active samples against acetylcholinesterase and glucosidase. All *Artemisia* samples displayed good antibacterial effects against *Mycobacterium tuberculosis* H37Ra, with MIC values of 64–256 mg/L. In conclusion, the investigated *Artemisia* species proved to be rich sources of bioactives endowed with antioxidant, enzyme inhibitory and anti-mycobacterial properties.

## 1. Introduction

*Artemisia* L. is a genus of small herbs and shrubs belonging to the Asteraceae family which inhabit the northern temperate regions of Asia, Europe and North America [1]. The *Artemisia* genus comprises over 500 species with significant medicinal and economic attributes due to their biological and chemical diversity [2]. *Artemisia* species are recognized for their characteristic strong aromas and bitter tastes, which are assigned to the presence of terpenes and sesquiterpene lactones [3]. Nonetheless, other classes of phenolic compounds, such as flavonoids, phenolic acids and coumarins, have been identified in various phytochemical studies [4,5]. Their aerial parts have a longstanding traditional use and are employed in the treatment of various ailments, including digestive disorders, inflammatory diseases, bronchitis, malaria, hepatitis and malignant diseases [5,6,7]. Over the past decades, the genus has attracted increasing attention in the field of drug discovery and development; many studies have unveiled its pleiotropic pharmacological profile, which includes anthelmintic, antimalarial, antitubercular, antiviral, antihyperlipidemic, antiemetic, antidepressant, anticancer, antiasthmatic, antihypertensive, antidiabetic, anxiolytic, hepatoprotective, gastroprotective and insecticidal effects [6,8,9,10].

The genus *Artemisia* is represented by 34 species in the flora of Romania [11], among which *A. absinthium* L., *A. annua* L., *A. austriaca* Jacq., *A. pontica* L. and *A. vulgaris* L. *A. absinthium* L., wormwood, are the best known species, with a wide distribution throughout Europe, North Africa, the Middle East and Asia. Wormwood is an ornamental and medicinal plant and has been used since antiquity as a bitter tonic, choleretic, anthelmintic and wound-healing agent [12]. The aerial parts contain essential oil (up to 1.5%, with marker compounds such as α- and β-thujone, thujyl alcohol, guaiazulene, (Z)-epoxyocimene, sabinyl acetate and chrysantenyl acetate), sesquiterpene lactones (absinthin and its isomers), flavonoids (apigenin, kaempferol, quercetin, artemethin and rutin), phenolic acids (caffeic, chlorogenic, ferulic, gallic, syringic and vanillic acids), coumarins (coumarin and herniarin), tannins, lignans, carotenoids, fatty acids and resins [1,10,12]. To date, numerous studies have unveiled other important bioactivities of *A. absinthium* aerial parts, e.g., antibacterial, antifungal, antiprotozoal, analgesic, anti-inflammatory, gastroprotective, hepatoprotective, neuroprotective, antidepressant and immunomodulatory properties [12,13,14]. Moreover, alongside its longstanding use in the alcoholic drinks industry (i.e., in the making of vermouth-type wines and absinthe), its current range of applications has rapidly expanded into the cosmetic and food industries [10].

*A. annua* L., sweet wormwood, is a herbaceous species that inhabits the temperate regions of Asia, Europe, Northern and Southern America, and Australia. The leaves and aerial parts have been traditionally used in Chinese and Hindu medicines as antipyretic agents in the treatment of malaria, tuberculosis and bacterial dysentery, and in the treatment of wounds, hemorrhoids and autoimmune diseases [8]. With the isolation in 1971 of the sesquiterpene lactone artemisinin as the active principle of *A. annua* against malaria, the species has received an increased interest from the scientific community [15]. Both artemisinin and its semi-synthetic derivatives artemether, arteether and artesunate have been employed clinically in the prophylaxis and treatment of malaria [16]. Alongside sesquiterpene lactones (e.g., artemisinin, arteannuins A–O, artemisitine and artemisinic acid), the aerial parts contain essential oil (up to 4%, with artemisia ketone, cadinene, camphene, camphor, β-caryophyllene and β-pinene as marker compounds), flavonoids (artemetin, casticin, derivatives of apigenin, kaempferol, isorhamnetin, luteolin and quercetin), phenolic acids (caffeic, rosmarinic, quinic and chlorogenic acids), coumarins (coumarin, esculetin, isofraxidine, melilotoside, tomentin, scopoletin and scopolin), polyalkenes, tannins, saponins, phytosterols and fatty acids [7,15,17,18]. Over the past decades, pharmacological studies have confirmed the known traditional uses of *A. annua* but have also unraveled novel bioactivities, such as anti-inflammatory, analgesic, anticancer, antihypertensive, antimicrobial, antioxidant and nephroprotective properties [17,19,20,21].

*A. austriaca* Jacq., Austrian wormwood, is a perennial herb found in the semi-arid lands of Central and Eastern Europe, Russia, Iran, Turkey and Northern China [22]. Phytochemical screening studies of aerial parts of *A. austriaca* have revealed the presence of essential oil (up to 1.1%, mainly comprising camphor, 1,8-cineole and camphene) [10,23], sesquiterpene lactones (arborescin, austricin, hydroxyachillin, artausin, matricarin and santonin) [22,24], flavonoids (cirsilineol, hesperidin, rutin, quercetin and luteolin-7-glycoside) [24,25,26] and phenolic acids (caffeic, chlorogenic and isochlorogenic acids) [27]. *A. austriaca* herbal extracts are traditionally used for their wound healing, choleretic, anthelmintic, anticonvulsant, anti-inflammatory and hemostatic activities [22,28]. Several studies have unveiled other important pharmacological properties, including antibacterial [29,30], antifungal [24,27,29,31] and antimalarial activities [24].

*A. pontica* L., Roman wormwood, is a perennial species distributed throughout Southeastern Europe, Siberia and Central Asia [8]. Compared to the allied *Artemisia* species, the phytochemical profile of *A. pontica* herbal extracts has not been comprehensively investigated, though several studies have reported the presence of sesquiterpene lactones (artemin, hydroxytaurin and hydroxyeudesmanolides) [8], essential oil (up to 2%, with 1,8-cineole, camphor, artemisia ketone and α-thujone as the main constituents) [1,32,33] and flavonoids (genkwanin and methyl esters of apigenin) [34]. *A. pontica* aerial parts are traditionally used as a bitter tonic and as sedatives and anthelmintics [33], but recent studies have proved additional beneficial effects, such as anti-inflammatory, analgesic [35], antioxidant [36] and insecticidal properties [37].

*A. vulgaris* L., common mugwort, is a species growing in the temperate and cold-temperature regions of Asia, Europe and North America, and has been employed as a culinary and medicinal herb [2]. The aerial parts have been traditionally used as a bitter tonic and anti-flatulent in treating gastrointestinal disorders and to alleviate gynecological ailments, such as amenorrhea or dysmenorrhea [38]. The main phytochemicals found in *A. vulgaris* include essential oil (up to 0.3%, comprising 1,8-cineole, sabinene, β-thujone and caryophyllene oxide as the main constituents), sesquiterpene lactones (vulgarin, psilostachyin and psilostachyin C), flavonoids (derivatives of kaempferol and quercetin), coumarins (coumarin, esculin, scopoletin and umbelliferone), phenolic acids (caffeic and chlorogenic acids), sterols, carotenoids and polyacetylenes [4,9,17]. To date, studies on *A. vulgaris* have confirmed its known traditional uses and revealed novel significant biological properties, e.g., antioxidant, spasmolytic, antibacterial, antifungal, antinociceptive, hepatoprotective, estrogenic and cytotoxic effects [2,17].

Our study aimed at promoting interest in the Romanian *Artemisia* species by providing novel insights into their metabolite profiles and bioactivities. To date, most studies have focused on the valorization of the aboveground parts with respect to their analgesic [35], anti-inflammatory [35,39], antimicrobial [40,41,42], antioxidant and cytotoxic properties [39,43,44]. To the best of our knowledge, we report herein for the first time a comprehensive phytochemical characterization of both the roots and aerial parts of five *Artemisia* spp. (*A. absinthium*, *A. annua*, *A. austriaca*, *A. pontica* and *A. vulgaris*) from the spontaneous flora of northeastern Romania by means of liquid chromatography–tandem high-resolution mass spectrometry (LC-HRMS/MS). The biological profile screening was achieved by in vitro testing of antioxidant (free radical scavenging, metal chelating and reducing power, and total antioxidant capacity), enzyme inhibitory (anti-cholinesterase, anti-tyrosinase, anti-amylase and anti-glucosidase) and anti-*Mycobacterium* activities.

## 2. Materials and Methods

### 2.1. Plant Materials and Preparation of Extracts

The aerial parts of the investigated *Artemisia* species were collected during August–September 2020, while the roots were collected during November–December 2020 from Neamt and Iasi counties, Romania, as follows: *A. absinthium*—Secuienii Noi (Neamt county, GPS coordinates: 46.843800, 26.887132), *A. annua*—Carlig (Iasi county, GPS coordinates: 47.195154, 27.567940), *A. austriaca*—Hadambu (Iasi county, GPS coordinates: 47.014004, 27.440596), *A. pontica*—Carlig (Iasi county, GPS coordinates: 47.200588, 27.562738), *A. vulgaris*—Secuienii Noi (Neamt county, GPS coordinates: 46.8408833, 26.8903426). The plant material was collected and authenticated by one of the authors (A.T.) and Dr. Constantin Mardari, Botanic Garden *Anastasie Fatu*, Iasi, Romania. Voucher specimens (AABH/2020, AANH/2020, AAUH/2020, APH/2020, AVH/2020, AABR/2020, AANR/2020, AAUR/2020, APR/2020 and AVR/2020) were deposited in the Department of Pharmacognosy, *Grigore T. Popa* University of Medicine and Pharmacy Iasi, Romania. The plant materials (aerial parts and roots collected from the investigated *Artemisia* spp.) were dried and ground and then 10 g was separately extracted with methanol and chloroform (100 mL) by ultrasonication (3 cycles of 30 min each, at room temperature). The obtained extracts were evaporated to dryness under vacuum (with the yields shown in Table 1) and kept at −20 °C until further analysis.

### 2.2. Total Phenolic and Flavonoid Content

Total phenolic content (TPC) and total flavonoid content (TFC) were determined as previously described [45,46], with the data provided as mg gallic acid equivalents (GAE)/g extract (TPC) and mg rutin equivalents (RE)/g extract (TFC), respectively.

### 2.3. LC-HRMS/MS Analysis

The liquid chromatography–tandem high-resolution mass spectrometry (LC-HRMS/MS) analysis of the methanol and chloroform extracts obtained from the roots and aerial parts of the five *Artemisia* species was carried out on an Agilent 1200 HPLC system (Agilent Technologies, Santa Clara, CA, USA) equipped with auto-sampler (G1329B), degasser (G1379B), binary pump (G1312C), thermostat (G1316A) and ESI-Q-TOF mass spectrometer (G6530B). The chromatographic separations were performed as follows: Phenomenex Gemini C18 column (2 mm × 100 mm, 3 μm); mobile phase 0.1% formic acid in water (A) and 0.1% formic acid in acetonitrile (B); gradient 5–60% B (0–45 min), 95% B (46–55 min); flow rate 0.2 mL/min; injection volume 10 μL. The following MS parameters were used: negative ionization mode; *m*/*z* range 100–1000; gas (N_2_) temperature 275 °C; N_2_ flow 10 L/min; nebulizer 35 psi; sheath gas temperature 325 °C; sheath gas flow rate 12 L/min; capillary voltage 4000 V; nozzle voltage 1000 V; skimmer 65 V; fragmentor 140 V; collision-induced dissociation energies 10 and 30 V.

### 2.4. Antioxidant and Enzyme Inhibitory Activity

The 1,1-diphenyl-2-picrylhydrazyl (DPPH) and 2,2′-azino-bis (3-ethylbenzothiazoline) 6-sulfonic acid (ABTS) radical scavenging, cupric ion reducing antioxidant capacity (CUPRAC), ferric ion reducing antioxidant power (FRAP), metal chelating ability (MCA) and phosphomolybdenum (PBD) assays were performed as previously detailed [45,46]. The results were expressed as mg Trolox equivalents (TE)/g for DPPH, ABTS, CUPRAC and FRAP assays, mg EDTA equivalents (EDTAE)/g for the MCA assay and mmol TE/g extract for the PBD assay. The inhibition assays against acetylcholinesterase (AChE), butyrylcholinesterase (BChE), tyrosinase, amylase and glucosidase were caried out as previously described [45,46]. The results were provided as mg galanthamine equivalents (GALAE)/g extract in the AChE and BChE assays, mg kojic acid equivalents [47]/g extract in the tyrosinase assay and mmol acarbose equivalents (ACAE)/g extract in the amylase and glucosidase assays. 

### 2.5. Anti-Mycobacterium Activity

#### 2.5.1. Inoculum Preparation

*Mycobacterium tuberculosis* H37Ra (ATCC 25177) was grown for two weeks on Löwenstein-Jensen slopes. The collected bacteria were transferred to 7H9-S medium (Middlebrook 7H9 broth supplemented with 10% ADC (albumin–dextrose–catalase) and 0.2% glycerol and vortexed with glass beads (1 mm diameter) for three minutes. After 30 min of room temperature incubation for larger clump sedimentation, the upper phase was transferred to a sterile tube and left for the second sedimentation for 15 min. Next, planktonic bacteria from above the sediment were placed in a fresh tube, with the turbidity adjusted to 0.5 McFarland standard with 7H9-S broth.

#### 2.5.2. MIC Determination

*Artemisia* extracts were tested in a concentration range of 256 to 16 mg/L. Serial twofold dilutions were prepared in dimethyl sulfoxide (DMSO) using a 7H9-S medium as dilution. The final DMSO concentration did not exceed 1% (*v*/*v*) and did not influence the growth of the tested strain. Ethambutol, rifampicin and streptomycin were used as reference standards. Stock solutions were prepared according to the manufacturer’s instructions. Final twofold dilutions from 16 to 0.001 mg/L were prepared in 7H9-S broth. The round bottom micro-well plates were prepared as follows: 50 μL of inoculum and 50 μL of tested substances were added to each well. The sterility, growth and 1% DMSO controls were included. The final density of the inoculum in each well was approximately 5 × 10^5^ CFU/mL. The plates were closed with sealing foil to prevent liquid evaporation and incubated for 8 days at 37 °C. Next, 10 µL of resazurin (Alamar Blue) solution was added to each well, followed by incubation for 48 h at 37 °C and assessment for color development. The minimum inhibitory concentration (MIC) was defined as the lowest drug concentration that prevented a blue to pink color change. The MIC determination was repeated twice. The obtained results were identical.

### 2.6. Data Analysis

A biological activities dataset was scaled, centered and submitted to principal component analysis (PCA) and hierarchical clustering analysis (HCA). For both PCA and HCA, “Ward’s rule” and “Euclidean distance” were employed for clustering. Afterwards, the biomolecules dataset was logarithm-transformed, scaled, centered and submitted to clustered image maps (CIMs). All multivariate analyses were performed using R v 4.1.2 software (R Foundation for Statistical Computing, Vienna, Austria). The Pearson correlation test was used to examine the relationship between phytoconstituents in tested extracts and biological activities. GraphPad. 9.0 (GraphPad Software, San Diego, CA, USA) was used for the correlation analysis.

## 3. Results and Discussion

### 3.1. Total Phenolic and Flavonoid Content

The TPCs and TFCs of the *Artemisia* extracts were determined using colorimetric methods. The results are given in Table 1. Apparently, methanol extracts contained more phenolics and flavonoids than chloroform in both plant parts. In addition, with the exception of *A. pontica*, the extracts of the aerial parts were richer than those of the roots in terms of total phenolics. The highest level of phenolics were determined in the methanol aerial part extracts of *A. vulgaris*, with 106.34 mg GAE/g. After that, the methanol aerial part extracts of *A. pontica* and *A. annua* contained significant levels of total phenolics (>50 mg GAE/g). Among the root extracts, the methanol extract from *A. annua* reached the highest value with 76.34 mg GAE/g, followed by the methanol extracts from *A. pontica* (65.65 mg GAE/g) and *A. austriaca* (41.68 mg GAE/g). With regard to the TFCs, the highest content was determined in the methanol extract *of A. annua* aerial parts with 47.74 mg RE/g, followed by the methanol extracts of *A. austriaca* (40.30 mg RE/g) and *A. vulgaris* (39.39 mg RE/g) aerial parts. The lowest level of total flavonoids was found in the chloroform extract of *A. absinthium* (0.37 mg RE/g). A number of studies have shown a comparable total content of phenolics and flavonoids in the *Artemisia* genus. For example, in a previous study, Ali et al. [48] found that the total phenolic content in *A. absinthium* extract was 3.61 mg GAE/g extract, which was lower than our values. In addition, Guo et al. [49] showed that the TPC and TFC in the aqueous extract of *A. annua* were 39.58 mg GAE/g and 7.04 mg RE/g, respectively. Our findings are also comparable to the results in the literature for other *Artemisia* species, such as *A. copa* (155.6 mg GAE/g dry plant in infused extract, reported by Larrazábal-Fuentes et al. [50]), *A. vulgaris* (117.14 mg GAE/g extract in methanol extract, reported by Jakovljevic et al. [51]), *A. alba* (110.20 mg GAE/g extract in methanol extract, reported by Jakovljevic et al. [51]) and *A. argy* (108.56 mg GAE/g extract in methanol extract, reported by Xiao et al. [52]). Although the spectrophotometric methods are widely used in phytochemical studies, recently, most phytochemists have been more concerned with colorimetric methods for assessing bioactive components [53]. This could be explained by the complex nature of phytochemicals and the fact that only specific compounds do not reduce the reagents used in the relevant assays. Given these facts, further chromatographic techniques are needed to evaluate the chemical profiles of plant extracts.

### 3.2. LC-HRMS/MS Analysis

The methanol and chloroform extracts obtained from the roots and aerial parts of the five *Artemisia* species were subsequently subjected to an in-depth LC-HRMS/MS analysis. The assignment of the peaks observed in the base peak chromatograms (BPCs) of the extract samples was performed by comparing the spectrometric data with the relevant literature [54,55,56,57,58,59,60,61] or online databases (KNApSacK; METLIN; NIST Chemistry WebBook). The metabolite profiling allowed the annotation of 73 compounds belonging to different phytochemical classes, such as phenolic acids, flavonoids, sesquiterpenes, organic acids, sugars, coumarins, triterpenes, lignans and fatty acids (Table 2, Table S1). In the following sub-sections, a brief description of these categories will be provided, whereas the intra- and interspecies differences will be thoroughly detailed in the Multivariate Analysis Section.

Out of the 15 phenolic acids identified in the *Artemisia* extracts, 2 were hydroxybenzoic acid derivatives (**3** and **4**), whereas the remaining were hydroxycinnamic acid derivatives. Of these, chlorogenic acid (**9**) was confirmed by comparing with the standard, whereas its isomers, neochlorogenic (**5**) and cryptochlorogenic (**8**) acids, were tentatively assigned based on their specific HRMS/MS fragments ions described in the literature [58,59]. Chlorogenic acid was identified in all five *Artemisia* species, especially in the aerial part extracts, whereas the other two isomers were present in all species, except for *A. annua*. In addition, several other quinic acid congeners were noticed, such as dicaffeoylquinic acids (**18**, **30** and **32**), feruloylquinic acid (**16**), coumaroylquinic acid (**19**), coumaroylcaffeoylquinic acid (**33**) and feruloylcaffeoylquinic acids (**35** and **38**). Compounds **18**, **19** and **33** were noticed only in *A. vulgaris*, whilst the ferulic acid derivatives (**16**, **35** and **38**) were present in *A. absinthium* and *A. annua*. Lastly, two glycosides of caffeic acid (**14**) and coumaric acid (**23**) were identified as specific metabolites in *A. vulgaris* (Table 2, Table S1). 

Flavonoids were the representative category of phytochemicals, with 26 different derivatives present exclusively in the extracts obtained from the aerial parts of *Artemisia*. Formally, they were grouped into free aglycones, *O*-glycosides and *C*-glycosides. Besides luteolin (**41**), identified based on standard injection, and eriodictyol (**39**), the other aglycones were tentatively identified as poly-hydroxylated/poly-methoxylated flavone derivatives. For instance, eupatolitin (**42**) was annotated only in *A. annua*; rhamnetin (**43**), diosmetin (**53**) and genkwanin (**65**) were characteristic of *A. pontica*; homoeriodictyol (**48**) was present only in *A. austriaca*; whereas eupalitin (**59**) and eupatilin (**68**) were spotted only in *A. vulgaris*. On the other hand, dihydroxytrimethoxyflavone (**52**) and casticin (**61**) were absent in *A. austriaca* and *A. pontica*, respectively. Cirsimaritin (**56**) and penduletin (**57**) were characteristic of both *A. austriaca* and *A. pontica*, while rhamnazin (**50**) was specific to *A. austriaca*, *A. pontica* and *A. vulgaris*. With respect to the *O*-glycosides, the following structures were tentatively proposed: mearnsetin-di-*O*-hexoside (**12**) in *A. annua*, quercetin-di-*O*-hexoside (**22**) in *A. austriaca*, luteolin-*O*-deoxyhexoside-*O*-hexoside (**28**) and rhamnetin-*O*-hexoside (**37**) in *A. vulgaris*, eupatolitin-*O*-deoxyhexoside-*O*-hexoside (**29**) in *A. absinthium* and eupatolitin-di-*O*-hexoside (**34**) and rhamnetin-di-*O*-hexoside (**36**) in *A. pontica*. On the other hand, quercetin-*O*-deoxyhexoside-*O*-hexoside (**25**) and mearnsetin-*O*-hexoside (**26**) were absent in *A. austriaca*, whilst quercetin-*O*-hexoside (**27**) was not present in *A. annua*. Lastly, the two apigenin-*C*-hexoside-*C*-pentosides (**21** and **24**) were specifically observed in *A. annua* (Table 2, Table S1).

A number of 14 sesquiterpenes have been identified in the aerial part extracts of *Artemisia* species; besides artemisinin (**40**), confirmed by standard injection, the other structures were proposed strictly in a tentative manner (Table 2, Table S1). Artemisinin (**40**) as well as deoxyartemisinins (**45** and **47**), pseudosantonin (**54**), artemisin C (**58**), arteannuin B (**60**), dihydroarteannuin B (**64**) and dihydrosantamarin (**66**) were specifically noticed in *A. annua*. Chrysartemins A (**13**) and B (**15**) were the only two sesquiterpenes in *A. austriaca*, whereas artabsinolide A (**17**) was the sole congener in *A. absinthium*. Artecanin hydrate (**20**) and cnicin (**62**) were characteristic of *A. pontica*, whilst santonin (**46**) was found exclusively in *A. vulgaris*.

Quinic acid (**1**) and sucrose (**2**) were two non-specific metabolites identified in all five *Artemisia* species. On the contrary, the five triterpenes, namely, absinthin (**55**), artenolide (**63**), isoabsinthin (**70**) and two absinthin derivatives (**67** and **71**), were characteristically noticed in *A. absinthium*. Three coumarins, such as esculetin (**7**) and two of its hexosides (**6** and **11**), were observed in *A. vulgaris*; additionally, one of the two esculetin-*O*-hexosides was also present in the roots of *A. austriaca*. Tracheloside, a glycosylated lignan, was putatively labeled in the aerial parts of *A. annua*, *A. austriaca* and *A. vulgaris*. Lastly, seven oxygenated fatty acids were assigned: trihydroxyoctadecenoic acid I (**49**), hydroxyoctadecatrienoic acid (**72**), hydroxyoctadecadienoic acid (**73**) and tuberonic acid-*O*-hexoside (**10**) in all *Artemisia* species; trihydroxyoctadecadienoic acid (**44**) in *A. absinthium*, *A. austriaca* and *A. vulgaris*; trihydroxyoctadecenoic acid II (**51**) in *A. absinthium* and *A. vulgaris*; and hydroperoxyoctadecadienoic acid (**69**) in *A. annua* and *A. austriaca* (Table 2, Table S1).

### 3.3. Antioxidant Activity

Antioxidant compounds are of increasing interest in the pharmaceutical and nutraceutical fields. These compounds provide powerful shields against free radicals, and a negative correlation between their consumption and the prevalence of chronic and degenerative diseases has been reported. In this sense, phytochemicals are considered a great treasure trove of antioxidants, and many compounds found in plants have been identified as natural and safe antioxidants. In the light of these facts, attempts were made to determine whether the tested *Artemisia* species are a source of natural antioxidants. Various chemical assays were performed, including radical quenching (ABTS and DPPH), reducing power (CUPRAC and FRAP), metal chelation and phosphomolybdenum assays. The results are shown in Table 3. Non-biological radicals, such as DPPH and ABTS, are commonly used in in vitro experiments to assess the abilities of plant extracts to scavenge radicals. From Table 3, the methanol extracts showed stronger radical scavenging abilities than the chloroform extracts in both parts. The best radical scavenging ability was found in the methanol extract of *A. annua* roots (DPPH: 237.03 mg TE/g; ABTS: 240.78 mg TE/g), followed by the methanol extracts of *A. pontica* roots (DPPH: 179.63 mg TE/g; ABTS: 176.12 mg TE/g) and *A. vulgaris* aerial parts (DPPH: 139.56 mg TE/g; ABTS: 173.86 mg TE/g) in both assays. The weakest radical scavenging ability was recorded in the chloroform extract of *A. absinthium* roots (DPPH: 5.11 mg TE/g; ABTS: 7.54 mg TE/g). The term “reducing power” refers to the ability of antioxidant compounds to donate electrons. For this purpose, CUPRAC and FRAP assays involving the conversion of Cu^+2^ to Cu^+^ and Fe^+3^ to Fe^+2^, respectively, were performed. In both plant parts, the methanol extracts had higher reducing potentials than the chloroform extracts. The methanol extracts of *A. vulgaris* aerial parts (498.32 mg TE/g), *A. annua* roots (438.43 mg TE/g) and *A. pontica* aerial parts (290.14 mg TE/g) exhibited the highest CUPRAC activities. With one small exception, the methanol extracts of *A. annua* roots (294.52 mg TE/g), *A. vulgaris* aerial parts (198.51 mg TE/g) and *A. pontica* roots (165.55 mg TE/g) demonstrated the highest levels of capability in the FRAP assay. The obtained results from the free radical scavenging and reducing power assays are almost consistent with the total phenolic results for the extracts. In this sense, the phenolic components in the extracts can be considered as the main contributors to the free radical scavenging and reducing abilities. Similar to our findings, several researches [62,63] reported a strong correlation between total phenolic content and antioxidant properties. However, we observed different results for metal chelation abilities. The chelating abilities of plant extracts may reflect the inhibition of hydroxyl radicals’ production in the Fenton reaction. The best metal chelating ability (MCA) was found in the methanol extract of *A. pontica* roots with 22.93 mg EDTAE/g extract. Intriguingly, in three *Artemisia* species tested, the chloroform extracts from the aerial parts had higher potentials than the methanol extracts (*A. annua, A. pontica* and *A. vulgaris*). In addition, two chloroform extracts of the roots (*A. annua* and *A. vulgaris*) showed no metal chelating ability. Non-phenolic chelators, such as polysaccharides, peptides or sulfides, may be responsible for the conflicting results. In support of our findings, several investigators reported a weak correlation between total phenolic content and metal chelating ability [64,65]. Some researchers also pointed out that the chelating ability of phenolics contributes only in a small extent to the antioxidant properties of plant extracts [66]. The phosphomolybdenum assay is related to the reduction of Mo (VI) to (Mo (V) by antioxidant compounds and is considered one of the total antioxidant capacity assays. As can be seen in Table 3, we observed different results for each species. For example, the aerial part extracts from two *Artemisia* species (*A. absinthium* and *A. vulgaris*) exhibited greater potentials than root extracts. In addition, the root extracts from two species (*A. annua* and *A. pontica*) showed stronger activity as compared to aerial parts. In the literature, several researchers have reported that the results from phosphomolybdenum assays exhibited weak correlations with total phenolic content [67,68]. This fact could be explained by the presence of non-phenolic antioxidants, such as tocopherol, ascorbic acid and terpenoids. *Artemisia* members have been previously found to possess interesting antioxidant properties. For example, in a recent study by Minda et al. [69], the DPPH radical scavenging abilities of three *Artemisia* species (*A. absinthium, A. dracunculus* and *A. annua*) were investigated, the materials exhibiting more than 90% scavenging ability at a concentration of 1000 µg/mL. In another study conducted by Kamarauskaite et al. [70], the fractions of *A. absinthium* and *A. ludoviciana* were assessed by ABTS and FRAP assays and their values were found to be 367–1693 µM TE/g and 5385–6952 µM TE/g, respectively. Ferrante et al. [71] also investigated the antioxidant properties of *A. santonicum* methanol extract (DPPH: 278.57 mg TE/g; ABTS: 217.60 mg TE/g; CUPRAC: 515.30 mg TE/g; FRAP: 255.35 mg TE/g; metal chelating: 21.96 mg EDTAE/g and phosphomolybdenum: 2.20 mmol TE/g). Other examples of *Artemisia* species whose antioxidant capacities have been determined in the literature include *A. lactiflora* [47], *A. indica* [72], *A. santolinifolia* [73] and *A. monosperma* [74]. Based on the solvents, plant parts, and species triangle, our results may provide new information on the antioxidant properties of *Artemisia* species.

### 3.4. Enzyme Inhibitory Activity

Enzyme inhibition is a concept that is currently gaining traction in the treatment of various global health problems, such as type 2 diabetes, obesity and Alzheimer’s disease. This phenomenon demonstrates that the inhibition of specific enzymes can be a highly effective therapeutic strategy to alleviate disease symptoms [75]. Amylase and glucosidase, for example, are thought to be important players in the management of blood glucose levels in diabetics [76]. Furthermore, acetylcholinesterase inhibitors may improve memory function in Alzheimer’s patients by increasing acetylcholine levels in the synapses [77]. Enzyme inhibitors, therefore, are being sought as a safe and effective way to treat the diseases listed above. In this sense, plants are considered excellent treasures [78]. Given these facts, we looked into the tested *Artemisia* species’ enzyme inhibitory properties. The results are summarized in Table 4. The best AChE inhibition was determined in the methanol extract of *A. absinthium* with 3.02 mg GALAE/g, followed by the chloroform extracts of *A. absinthium* (2.50 mg GALAE/g) and *A. annua* (2.36 mg GALAE/g) aerial parts. With regard to BChE inhibition, the chloroform root extracts (*A. annua*, *A. austriaca* and *A. absinthium*) were recorded as the strongest extracts. With the exception of *A. vulgaris* roots, the chloroform extracts were more active against BChE than the methanol extracts in all tested *Artemisia* species. Two methanol root extracts (*A. austriaca* and *A. pontica*) were not active on BChE. As can be seen from Table 4, tyrosinase inhibitory effects were higher in the methanol extracts as compared to the chloroform extracts, except for *A. vulgaris* aerial parts. The most active methanol extracts were *A. annua* (49.42 mg KAE/g), *A. austriaca* (47.27 mg KAE/g) and *A. pontica* (44.91 mg KAE/g). The weakest tyrosinase inhibition potential was found in the chloroform extract of *A. austriaca* roots with 13.16 mg KAE/g. For all parts and species, the chloroform extracts had stronger amylase inhibitory effects than the methanol extracts. The best amylase inhibitory effects were recorded in the chloroform extracts of *A. austriaca* parts (root: 0.57 mmol ACAE/g; aerial parts: 0.54 mmol ACAE/g). As for glucosidase inhibitory activity, the aerial parts of all *Artemisia* species showed stronger abilities than the root extracts, and the best ability was obtained by the methanol extract of *A. vulgaris* aerial parts (11.32 mmol ACAE/g). The methanol root extract of *A. austriaca* had the weakest glucosidase inhibitory effect (0.16 mmol ACAE/g). To the best of our knowledge, scientific information on enzyme inhibitory properties of the members of the genus *Artemisia* is scarce [56,71,79,80,81,82,83]. In this sense, the present work could provide further insights into the future application of *Artemisia* species as natural sources of enzyme inhibitory agents.

### 3.5. Anti-Mycobacterium Activity

The emergence of multidrug-resistant *Mycobacterium tuberculosis* strains represents a major barrier to tuberculosis eradication, leading to longer treatment regimens, higher toxicity and even treatment failure [84]. Thus, there is an urgent demand to explore novel drugs and combinations to improve tuberculosis therapy. Recent work has presented the antimalarial drug artemisinin as a promising antitubercular agent [85,86]. Moreover, Martini et al. found that dichloromethane extracts from leaves of *A. annua* L. and *A. afra* Jacq. ex Willd. displayed even higher anti-mycobacterial effects than sesquiterpene artemisinin [87]. In the present study, *M. tuberculosis* H37Ra was exposed to chloroform and methanol extracts obtained from the roots and aerial parts of five *Artemisia* species. Within the tested concentration range, most of the samples were active against the mycobacterial strain, except for the methanol root extracts of *A. absinthium, A. austriaca* and *A. vulgaris* (Table 5). The chloroform extract of *A. austriaca* aerial parts showed the highest anti-mycobacterial effect (MIC = 64 mg/L), while the other active extracts displayed similar degrees of potency, with MICs of 128–256 mg/L. Considering that plant extracts can be categorized as having strong activity when their MIC is within the range of 50–500 mg/L, moderate activity with an MIC of 500–1500 mg/L and weak activity with an MIC above 1500 mg/L [88], it can be stated that the *Artemisia* samples possess strong anti-*Mycobacterium* effects. To the best of our knowledge, previous studies referred only to the anti-mycobacterial potential of *Artemisia* herbal extracts. We report herein for the first time on the anti-mycobacterial activity of *Artemisia* root extracts and that their potency was found to be similar to that of the aerial part extracts. Our results are in agreement with the study of Bhowmick et al. [89] which showed the inhibitory effects of an hexane extract from *A. annua* aerial parts against *Mycobacterium smegmatis* (MIC range: 250–1000 mg/L). Further, the bioactivity-linked fractionation of the extract revealed that its inhibitory effects were due to the sesquiterpenes deoxyartemisinin and artemisinic acid [89]. Regarding our *Artemisia* species, the LC-MS/MS analysis showed that chrysartemin A and B were present only in the most active sample—*A. austriaca* herbal extract (Table S1). These two compounds, which were previously reported in other *Artemisia* species (*A. mexicana* and *A. klotzchiana*) [90], belong to the guaianolide-type sesquiterpenes known for their inhibitory effects against *Mycobacterium* strains [90,91]. Therefore, we can conclude that sesquiterpenes found in *Artemisia* extracts may contribute to their overall anti-mycobacterial activity; moreover, it cannot be excluded that the synergistic effects among different classes of constituents identified in our samples (e.g., sesquiterpenes, phenolic acids, flavonoids, coumarins, fatty acids) might explain the observed effects.

### 3.6. Multivariate Analysis

**Figure 1:**
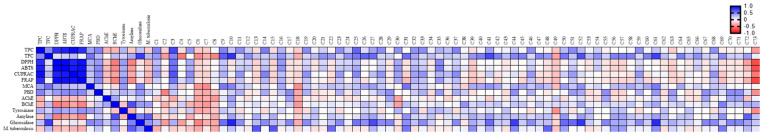
Correlation analysis of the phytochemical composition and biological activities. ABTS, 2,2′-azino-bis (3-ethylbenzothiazoline) 6-sulfonic acid; AChE, acetylcholinesterase; BChE, butyrylcholinesterase; CUPRAC, cupric ion reducing antioxidant capacity; DPPH, 1,1-diphenyl-2-picrylhydrazyl; FRAP, ferric ion reducing antioxidant power; MCA, metal chelating activity; PDA, phosphomolybdenum activity; TPAC, total phenolic acid content; TPC, total phenolic content. Compounds numbered as in Table 2.

A Pearson correlation analysis of bioactive compounds and biological activities was performed. The correlation heatmap is shown in Figure 1. Clearly, total phenolic content was highly correlated (R > 0.8) with scavenging and reducing abilities. However, the metal chelation and phosphomolybdenum assays correlated moderately with total phenolic levels. This fact could be explained by the presence of non-phenolic chelators (polypeptides, sulfides, etc.) or antioxidants (vitamin C, tocopherols, etc.). As can be seen in Figure 1, the individual compounds exhibited different correlations with the biological activities. Dihydroxybenzoic acid hexoside (**3**) and feruloylcaffeoylquinic acid I (**35**) were strongly correlated with free radical scavenging and reducing power abilities. However, different compounds exhibited a linear correlation in the metal chelation and phosphomolybdenum assays. The main players were dihydroxytetramethoxyflavone (**61**) and chlorogenic acid (**9**) in the metal chelation assay. In the phosphomolybdenum assay, artabsinolide A (**17**) and absinthin derivative II (**71**) were found to be the main contributors. With regard to the enzyme inhibition assays, different compounds acted as inhibiting agents in each assay. Tuberonic acid-*O*-hexoside (10) was moderately correlated with cholinesterases (AChE and BChE). For tyrosinase inhibition, dicaffeoylquinic acid derivatives (**30** and **32**) had low correlation values (R < 0.4). Dihydroxytrimethoxyflavone (52) and dihydroxytetramethoxyflavone (**61**) were closely associated with glucosidase inhibition assay (R > 0.7). Two sesquiterpenes (chrysartemin A (**13**) and B (**15**)) showed stronger correlations with anti-*Mycobacterium* ability compared to other compounds. Taken together, the tested extracts have great potential as natural sources of bioactive agents and could therefore be considered as valuable raw materials with pharmaceutical and nutraceutical applications.

**Figure 2:**
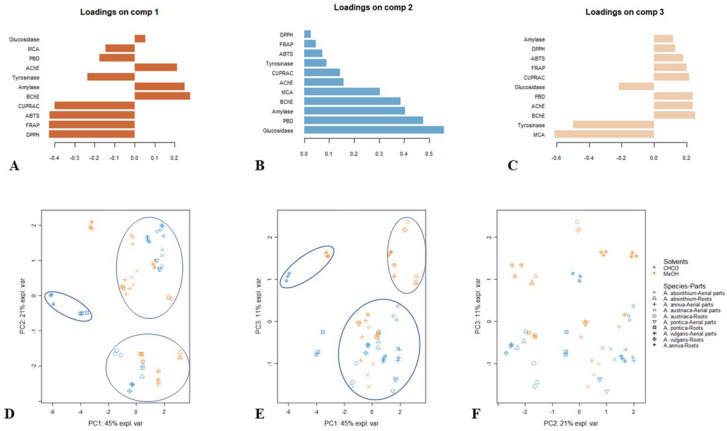
Exploratory principal component analysis. (**A**–**C**) Contribution of biological activities to the principal components of the PCA. (**D**–**F**) Scatter plot showing the distribution of the samples in the factorial plan derived from the three retained principal components.

**Figure 3:**
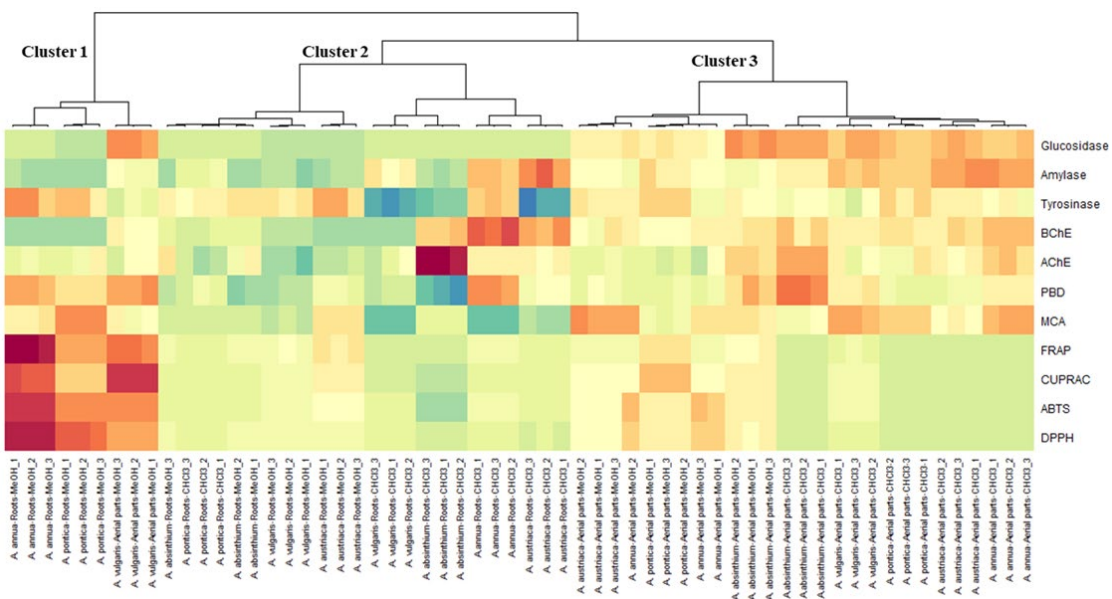
Clustered image map (red color: high bioactivity; blue color: low bioactivity) based on the biological activities dataset.

The results of the PCA for the antioxidant and enzyme inhibitory activities of *Artemisia* species are presented in Figure 2. Firstly, the screening of the eigenvalues suggested that the first three principal components (PCs) were sufficient to synthesize most of the data variation. Indeed, these components manifested a variance of 45%, 21% and 11%, respectively. The first PC represented the variation in the antioxidant activities, since it was predominantly and negatively linked with both radical scavenging (ABTS and DPPH) and reducing power (FRAP and CUPRAC) (Figure 2A). The second PC discriminated the samples based on their anti-glucosidase, phosphomolybdenum, anti-amylase and anti-BChE activities (Figure 2B), as it was significantly and positively bound to the mentioned bioactivities. The third PC separated the samples in terms of their metal chelating capacity and anti-tyrosinase activities (Figure 2C). Examination of the scatter plots (PC1 vs. PC2, PC1 vs. PC3 and PC2 vs. PC3) reported in Figure 2D–F evidenced considerable variability among the samples. Three groups seem to emerge in the first scatter plot (PC1 vs. PC2) (Figure 2D); the same trend is noticed in the second scatter plot (PC1 vs. PC3); however, the samples representing the three groups were different from those obtained previously (Figure 2E). In the third scatter plot (PC2 vs. PC3), no clear clusters were identified (Figure 2F). For a better identification of the different groups, HCA was applied by using the coordinates of the samples on the three dimensions of the PCA. By using Ward’s method and Euclidean distance, we obtained three main clusters (Figure 3). Among the clusters, the samples representing cluster 1 (i.e., the methanol root extracts of *A. annua*, *A. pontica* and aerial part extracts of *A. vulgaris*) were characterized by the highest radical scavenging (ABTS and DPPH) and reducing power (FRAP and CUPRAC) activities.

**Figure 4:**
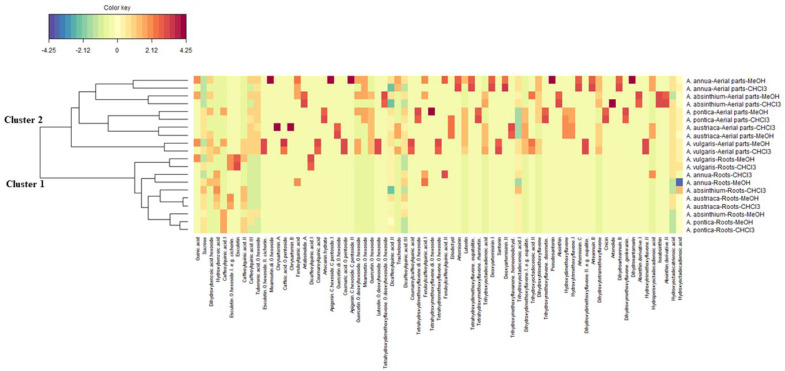
Clustered image map (red color: high bioactivity; blue color: low bioactivity) based on the chemical composition dataset.

Next, to determine the phytochemical differences between the studied *Artemisia* species, CIM analysis with respect to the phytochemical compounds dataset was carried out. The extracts were separated into two large clusters, namely, the roots on the one hand and the aerial parts on the other (Figure 4). Overall, several compounds were more abundant in the extracts from the aerial parts than in the extracts from the roots. Furthermore, in cluster 2, which represented all the aerial part samples, the methanol and chloroform extracts of all species were very similar. On the other hand, in cluster 2, *A. annua* extracts were similar to *A. absinthium* extracts and *A. pontica* extracts were similar to *A. austriaca* extracts, whereas the *A. vulgaris* aerial part extracts represented a distinct cluster. Similarly, in cluster 1, both *A. vulgaris* root extracts were found to be clearly different as compared to the other extracts. These findings suggest that *A. vulgaris* distinguished itself from the other four *Artemisia* species investigated in the current work. Moreover, some compounds of *A. vulgaris* (i.e., caffeic acid-*O*-pentoside, esculetin-*O*-hexoside I, coumaroylquinic acid, coumaric acid-*O*-pentoside, luteolin-*O*-deoxyheoside-*O*-hexoside and coumaroylcaffeoylquinic acid) could be used as potential markers for this species, due to their abundance in the aerial parts of *A. vulgaris*.

## 4. Conclusions

In this work, five *Artemisia* species collected from the spontaneous flora of northeastern Romania, namely, *A. absinthium*, *A. annua*, *A. austriaca*, *A. pontica* and *A. vulgaris*, were comprehensively investigated with respect to their phytochemical profiles and multi-biological potential (antioxidant, enzyme inhibitory and anti-mycobacterial). The LC-HRMS/MS-based metabolite profiling allowed the annotation of 73 different compounds, of which 15 were phenolic acids (i.e., chlorogenic, neochlorogenic, dicaffeoylquinic, feruloylquinic, coumaroylquinic acids), 26 were flavonoids (i.e., as poly-hydroxylated/poly-methoxylated flavones) and 14 were sesquiterpenes (i.e., artemisinin, pseudosantonin, arteannuin B). CIM analysis of the phytochemical profile revealed three main clusters, the first comprising *A. annua* together with *A. absinthium*, the second *A. pontica* together with *A. austriaca* and the third *A. vulgaris*. The antioxidant activity analysis of the five species revealed the superior antioxidant activity of the aerial part extracts as compared to the root extracts, as well as the better antioxidant activity of the methanol extracts as compared to the chloroform extracts. Furthermore, PCA and HCA allowed us to differentiate the samples into three main clusters with respect to antioxidant and enzyme inhibitory potential, with one cluster (cluster 1—the methanol root extracts of *A. annua* and *A. pontica* and the aerial part extracts of *A. vulgaris*) being characterized by the highest radical scavenging (ABTS and DPPH) and reducing power (FRAP and CUPRAC) activities. In addition, the chloroform extract of *A. austriaca* aerial parts showed the highest antibacterial effects against *M. tuberculosum* H37Ra (MIC = 64 mg/L), while other extracts displayed MIC values of 128–256 mg/L. Aside from the chemotaxonomic importance, the current study makes significant contributions to knowledge of the chemical and versatile biological profile of the investigated *Artemisia* ssp. collected from Romanian flora. Overall, our research could open prospects for the large-scale exploitation of *Artemisia* species (both roots and aerial parts) as rich sources of bioactive metabolites endowed with interesting antioxidant, enzyme inhibitory and anti-mycobacterial properties.

## Figures and Tables

**Table 1:** Extraction yields and total phenolic and flavonoid contents of *Artemisia* spp. extracts.

*Artemisia* Species	Part	Extraction Solvent	Yield (%)	TPC (mg GAE/g)	TFC (mg RE/g)
*A. absinthium* L.	Roots	MeOH	15.66	19.77 ± 0.20 ^f^	2.35 ± 0.04 ^e^
		CHCl_3_	5.79	5.78 ± 0.10 ^g^	0.37 ± 0.02 ^g^
	Aerial parts	MeOH	18.75	53.38 ± 0.16 ^d^	28.74 ± 0.51 ^d^
		CHCl_3_	9.47	18.28 ± 0.15 ^i^	24.25 ± 1.28 ^e^
*A. annua* L.	Roots	MeOH	2.19	76.35 ± 0.75 ^a^	10.41 ± 0.27 ^a^
		CHCl_3_	0.68	26.10 ± 0.10 ^d^	1.41 ± 0.07 ^f^
	Aerial parts	MeOH	17.57	60.00 ± 0.24 ^c^	47.74 ± 0.79 ^a^
		CHCl_3_	10.69	25.27 ± 0.20 ^g^	35.36 ± 0.30 ^c^
*A. austriaca* Jacq.	Roots	MeOH	11.73	41.68 ± 0.25 ^c^	4.65 ± 0.20 ^c^
		CHCl_3_	1.57	26.59 ± 0.23 ^d^	2.37 ± 0.01 ^e^
	Aerial parts	MeOH	13.88	48.42 ± 0.49 ^e^	40.30 ± 0.94 ^b^
		CHCl_3_	8.12	24.50 ± 0.14 ^g^	32.89 ± 1.58 ^c^
*A. pontica* L.	Roots	MeOH	6.55	65.65 ± 0.46 ^b^	6.99 ± 0.17 ^b^
		CHCl_3_	1.16	23.59 ± 0.58 ^e^	2.46 ± 0.03 ^e^
	Aerial parts	MeOH	22.95	65.06 ± 0.59 ^b^	33.01 ± 0.43 ^c^
		CHCl_3_	9.46	22.65 ± 0.18 ^h^	26.85 ± 1.02 ^de^
*A. vulgaris* L.	Roots	MeOH	12.45	27.36 ± 0.99 ^d^	3.29 ± 0.12 ^d^
		CHCl_3_	1.03	22.21 ± 0.82 ^e^	1.13 ± 0.17 ^f^
	Aerial parts	MeOH	15.67	106.34 ± 0.61 ^a^	39.39 ± 0.86 ^b^
		CHCl_3_	5.86	37.62 ± 0.09 ^f^	11.02 ± 0.78 ^f^

Data are presented as mean ± standard deviation (SD) of three determinations; different superscript letters within columns indicate significant differences in the tested extracts for the same parts (*p* < 0.05). GAE, gallic acid equivalents; RE, rutin equivalents; TFC, total flavonoid content; TPC, total phenolic content.

**Table 2:** LC-HRMS/MS-based phytochemical profiling of *Artemisia* spp. extracts.

No.	Proposed Identity	Class	T_R_ (min)	HRMS	Exp. (*m*/*z*)	Calcd. (*m*/*z*)	Δ (ppm)	HRMS/MS (*m*/*z*)
**1**	Quinic acid *	Organic acid	1.83	[M − H]^−^	191.0557	191.0561	2.14	173.0381, 127.0340, 111.0384
**2**	Sucrose	Sugar	1.86	[M − H]^−^	341.1097	341.1089	−2.24	179.0571, 119.0312
**3**	Dihydroxybenzoic acid hexoside	Phenolic acid	7.58	[M − H]^−^	315.0706	315.0722	4.92	153.0105, 109.0215
**4**	Hydroxybenzoic acid *	Phenolic acid	9.98	[M − H]^−^	137.0241	137.0244	2.30	109.0358
**5**	Neochlorogenic acid	Phenolic acid	10.27	[M − H]^−^	353.0893	353.0878	−4.22	191.0484, 179.0252, 135.0370
**6**	Esculetin-*O*-hexoside I	Coumarin	10.91	[M − H]^−^	339.0715	339.0722	1.93	177.0233, 149.0157, 133.0217, 105.0327
**7**	Esculetin	Coumarin	14.41	[M − H]^−^	177.0207	177.0193	−7.68	133.0227, 105.0266
**8**	Cyrptochlorogenic acid	Phenolic acid	15.71	[M − H]^−^	353.0880	353.0878	−0.55	191.0488, 173.0429, 161.0239, 135.0412
**9**	Chlorogenic acid *	Phenolic acid	16.69	[M − H]^−^	353.0893	353.0878	−4.22	191.0568, 173.0429, 135.0461
**10**	Tuberonic acid-*O*-hexoside	Fatty acid	17.48	[M − H]^−^	387.1681	387.1661	−5.27	207.1010, 163.1121, 119.0376
**11**	Esculetin-*O*-hexoside II	Coumarin	18.19	[M − H]^−^	339.0728	339.0722	−1.89	177.0203, 149.0144, 133.0215
**12**	Mearnsetin-di-*O*-hexoside	Flavonoid	18.57	[M − H]^−^	655.1575	655.1516	−1.39	493.1190, 331.0475, 315.0138
**13**	Chrysartemin A	Sesquiterpene	18.99	[M − H]^−^	277.1072	277.1081	3.41	233.1193, 218.0969, 215.1098, 191.1061, 175.0763, 135.0835
**14**	Caffeic acid-*O*-pentoside	Phenolic acid	19.50	[M − H]^−^	311.0767	311.0772	1.73	179.0343, 149.0461, 135.0440
**15**	Chrysartemin B	Sesquiterpene	20.38	[M − H]^−^	277.1066	277.1081	5.56	233.1165, 215.0981, 191.1034, 175.0705, 160.0463, 135.0839
**16**	Feruloylquinic acid	Phenolic acid	20.52	[M − H]^−^	367.1049	367.1035	−3.92	191.0563, 173.0460, 134.0349
**17**	Artabsinolide A	Sesquiterpene	20.60	[M − H]^−^	279.1237	279.1237	0.35	261.1027, 243.0906, 217.1121, 199.1051, 175.1082
**18**	Dicaffeoylquinic acid I	Phenolic acid	20.65	[M − H]^−^	515.1198	515.1195	−0.58	353.0763, 191.0485, 179.0265, 135.0373
**19**	Coumaroylquinic acid	Phenolic acid	20.67	[M − H]^−^	337.0926	337.0929	0.86	191.0589, 173.0462, 145.0322, 109.0380
**20**	Artecanin hydrate	Sesquiterpene	21.15	[M − H]^−^	295.1187	296.1187	−4.69	251.1300, 207.1409, 189.1280, 151.0831
**21**	Apigenin-*C*-hexoside-*C*-pentoside I	Flavonoid	21.33	[M − H]^−^	563.1404	563.1406	0.41	503.1277, 383.0784, 353.0680, 325.0671, 297.0714
**22**	Quercetin-di-*O*-hexoside	Flavonoid	21.51	[M − H]^−^	625.1418	625.1410	−1.24	463.0810, 300.0246, 271.0240, 151.0020
**23**	Coumaric acid-*O*-pentoside	Phenolic acid	21.81	[M − H]^−^	295.0819	295.0823	1.44	163.0416, 149.0463, 119.0494
**24**	Apigenin-*C*-hexoside-*C*-pentoside II	Flavonoid	22.12	[M − H]^−^	563.1414	563.1406	−1.37	443.1010, 383.0747, 353.0663, 325.0728, 297.0763
**25**	Quercetin-*O*-deoxyhexoside-*O*-hexoside	Flavonoid	23.15	[M − H]^−^	609.1476	609.1461	−2.44	300.0167, 271.0154, 150.994
**26**	Mearnsetin-*O*-hexoside	Flavonoid	23.91	[M − H]^−^	493.0998	493.0988	−2.10	331.0495, 315.0183, 287.0218, 271.0266
**27**	Quercetin-*O*-hexoside	Flavonoid	24.10	[M − H]^−^	463.0860	463.0882	4.74	300.0210, 255.0139, 150.9999
**28**	Luteolin-*O*-deoxyhexoside-*O*-hexoside	Flavonoid	24.79	[M − H]^−^	593.1540	593.1512	−4.72	285.0443, 255.0292, 227.0355, 151.0042
**29**	Eupatolitin-*O*-deoxyhexoside-*O*-hexoside	Flavonoid	25.01	[M − H]^−^	653.1729	653.1723	−0.88	345.0825, 330.0441, 301.0478, 287.0236
**30**	Dicaffeoylquinic acid II	Phenolic acid	25.92	[M − H]^−^	515.1198	515.1195	−0.58	353.0763, 191.0485, 179.0265, 135.0373
**31**	Tracheloside	Lignan	26.38	[M − H]^−^	549.1985	549.1978	−1.36	505.1054, 387.1727, 301.0335, 207.1026, 161.0258
**32**	Dicaffeoylquinic acid III	Phenolic acid	26.78	[M − H]^−^	515.1188	515.1195	1.36	353.0773, 191.0481, 179.0258, 173.0390
**33**	Coumaroylcaffeoylquinic acid	Phenolic acid	27.81	[M − H]^−^	499.1307	499.1246	−1.63	353.0922, 337.0981, 191.0566, 163.0440
**34**	Eupatolitin-di-*O*-hexoside	Flavonoid	27.98	[M − H]^−^	669.1645	669.1672	4.09	345.0667, 330.0402, 301.0186, 179.0381, 161.0253
**35**	Feruloylcaffeoylquinic acid I	Phenolic acid	28.31	[M − H]^−^	529.1397	529.1351	0.85	367.1386, 353.1172, 191.0748, 179.0486, 161.0397
**36**	Rhamnetin-di-*O*-hexoside	Flavonoid	28.95	[M − H]^−^	639.1527	639.1567	6.21	413.1265, 315.0608, 300.0298, 284.0403, 271.0291, 255.0388
**37**	Rhamnetin-*O*-hexoside	Flavonoid	29.02	[M − H]^−^	477.1016	477.1038	4.71	433.1382, 315.0767, 161.0276, 153.0227, 109.0304
**38**	Feruloylcaffeoylquinic acid II	Phenolic acid	29.12	[M − H]^−^	529.1353	529.1351	−0.28	367.1035, 353.0930, 191.0589, 179.0320, 173.0484
**39**	Eriodictyol	Flavonoid	29.39	[M − H]^−^	287.0567	287.0561	−2.04	151.0046, 135.0479
**40**	Artemisinin *	Sesquiterpene	30.44	[M − H]^−^	281.1385	281.1394	3.36	263.1319, 237.1529, 193.1612
**41**	Luteolin *	Flavonoid	31.04	[M − H]^−^	285.0400	285.0405	1.61	175.0386, 133.0313
**42**	Tetrahydroxydimethoxyflavone (e.g., eupatolitin)	Flavonoid	31.49	[M − H]^−^	345.0602	345.0616	4.02	330.0402, 315.0188, 287.0296, 259.0301, 259.0301, 215.0351, 175.0091, 149.0308, 121.0326
**43**	Tetrahydroxymethoxyflavone (e.g., rhamnetin)	Flavonoid	31.55	[M − H]^−^	315.0509	315.0510	0.40	300.0327, 271.0269, 255.0312, 243.0322, 227.0356, 215.0350, 171.0409, 147.0202
**44**	Trihydroxyoctadecadienoic acid	Fatty acid	31.79	[M − H]^−^	327.2181	327.2177	−1.43	229.1442, 211.1319
**45**	Deoxyartemisinin I	Sesquiterpene	32.09	[M − H]^−^	265.1435	265.1445	3.88	247.1335, 221.1582, 203.1459, 185.1346, 151.1148
**46**	Santonin	Sesquiterpene	32.42	[M − H]^−^	245.1174	245.1183	3.73	201.1282, 186.1064, 161.0962, 147.0805, 135.0841
**47**	Deoxyartemisinin II	Sesquiterpene	32.70	[M − H]^−^	265.1447	265.1445	−1.00	247.1357, 221.1557, 203.1451, 151.1154
**48**	Trihydroxymethoxyflavanone (e.g., homoeriodictyol)	Flavonoid	32.98	[M − H]^−^	301.0724	301.0718	−2.11	151.0049, 134.0413
**49**	Trihydroxyoctadecenoic acid I	Fatty acid	33.62	[M − H]^−^	329.2331	329.2333	0.75	229.1470, 211.1353, 199.1170
**50**	Dihydroxydimethoxyflavone I (e.g., rhamnazin)	Flavonoid	33.69	[M − H]^−^	329.0678	329.0667	−3.40	314.0456, 299.0241, 271.0279, 271.0272, 243.0312, 227.0430, 215.0360, 199.0421, 185.0236, 161.0264, 151.0068, 133.0347
**51**	Trihydroxyoctadecenoic acid II	Fatty acid	34.05	[M − H]^−^	329.2338	329.2333	−1.37	229.1433, 199.1155
**52**	Dihydroxytrimethoxyflavone	Flavonoid	34.72	[M − H]^−^	359.0772	359.0772	0.11	344.0575, 329.03351, 314.0086, 297.0051, 286.0162, 270.0287, 258.0184, 230.0225, 214.0302, 202.0280
**53**	Trihydroxymethoxyflavone (e.g., diosmetin)	Flavonoid	36.93	[M − H]^−^	299.0566	299.0561	−1.63	284.0259, 255.0179, 239.0292, 227.0330, 151.0077, 133.0252
**54**	Pseudosantonin	Sesquiterpene	36.96	[M − H]^−^	263.1279	263.1289	3.72	245.1127, 219.1366, 201.1230, 159.1152
**55**	Absinthin	Triterpene	37.16	[M + HCO_2_]^−^	541.2801	541.2807	1.19	351.6359, 275.5226
**56**	Hydroxydimethoxyflavone (e.g., cirsimaritin)	Flavonoid	37.25	[M − H]^−^	313.0711	313.0718	2.11	298.0722, 283.0375, 269.0628
**57**	Hydroxytrimethoxyflavone I (e.g., penduletin)	Flavonoid	37.67	[M − H]^−^	343.0813	343.0823	2.98	328.0382, 313.0382, 298.0133, 285.0421, 270.0199, 255.0318, 242.0284
**58**	Artemisinin C	Sesquiterpene	37.72	[M − H]^−^	247.1329	247.1340	4.30	231.1403, 203.1469, 187.1442, 161.1372, 133.1030
**59**	Dihydroxydimethoxyflavone II (e.g., eupalitin)	Flavonoid	37.99	[M − H]^−^	329.0678	329.0667	−3.40	314.0456, 299.0241, 271.0279, 271.0272, 243.0312, 227.0430, 215.0360, 199.0421, 185.0236, 161.0264, 151.0068, 133.0347
**60**	Arteannuin B	Sesquiterpene	38.36	[M − H]^−^	247.1341	247.1340	−0.53	203.1449, 133.1019
**61**	Dihydroxytetramethoxyflavone (e.g., casticin)	Flavonoid	38.81	[M − H]^−^	373.0939	373.0929	−2.70	358.0729, 343.0494, 300.0407, 285.0054, 269.0079, 257.0103, 241.0132, 229.0140, 213.0161, 201.0202, 185.0220
**62**	Cnicin	Sesquiterpene	39.38	[M − H]^−^	377.1617	377.1606	−2.97	295.1213, 251.1322, 189.1257, 151.07060
**63**	Artenolide	Triterpene	40.18	[M + HCO_2_]^−^	573.2714	573.2705	−1.66	527.2685, 325.1304, 263.1287, 185.1288
**64**	Dihydroarteannuin B	Sesquiterpene	40.28	[M − H]^−^	249.1496	249.1496	0.70	231.1415, 207.1742, 187.1523
**65**	Dihydroxymethoxyflavone (e.g., genkwanin)	Flavonoid	40.84	[M − H]^−^	283.0601	283.0612	3.86	268.0423, 240.0392, 211.0419
**66**	Dihydrosantamarin	Sesquiterpene	41.37	[M − H]^−^	249.1508	249.1496	−4.72	231.1471, 205.1599, 187.1494
**67**	Absinthin derivative I	Triterpene	41.96	[M + HCO_2_]^−^	555.2582	555.2600	3.44	509.2392, 491.2392, 447.2558, 265.1365, 243.1047, 229.1237, 199.1137
**68**	Hydroxytrimethoxyflavone II (e.g., eupatilin)	Flavonoid	37.67	[M − H]^−^	343.0813	343.0823	2.98	328.0382, 313.0382, 298.0133, 285.0421, 270.0199, 255.0318, 242.0284
**69**	Hydroperoxyoctadecadienoic acid	Fatty acid	44.54	[M − H]^−^	311.2212	311.2228	5.07	293.2171, 211.1341, 171.0999
**70**	Isoabsinthin	Triterpene	44.67	[M + HCO_2_]^−^	541.2801	541.2807	1.19	495.2583, 351.6359, 275.5226
**71**	Absinthin derivative II	Triterpene	46.17	[M + HCO_2_]^−^	539.2672	539.2650	−4.37	247.1212, 204.1637, 185.1479
**72**	Hydroxyoctadecatrienoic acid	Fatty acid	47.14	[M − H]^−^	293.2118	293.2122	1.42	275.1973, 224.1359, 195.1381
**73**	Hydroxyoctadecadienoic acid	Fatty acid	48.69	[M − H]^−^	295.2269	295.2279	3.27	277.2162, 195.1407, 171.1029

* Identified based on the standard.

**Table 3:** Antioxidant activity of *Artemisia* spp. extracts.

*Artemisia* Species	Part	Extraction Solvent	DPPH (mg TE/g)	ABTS (mg TE/g)	CUPRAC (mg TE/g)	FRAP (mg TE/g)	MCA (mg EDTAE/g)	PBD (mmol TE/g)
*A. absinthium* L.	Roots	MeOH	43.59 ± 1.08 ^c^	47.67 ± 0.34 ^f^	82.69 ± 1.76 ^e^	52.80 ± 2.52 ^e^	7.25 ± 0.23 ^e^	1.20 ± 0.11 ^e^
		CHCl_3_	5.11 ± 0.22 ^f^	7.54 ± 0.21 ^g^	24.24 ± 0.26 ^g^	12.40 ± 0.09 ^h^	8.33 ± 0.14 ^d^	0.84 ± 0.08 ^f^
	Aerial parts	MeOH	67.57 ± 3.55 ^cd^	95.95 ± 3.61 ^c^	188.11 ± 5.68	85.36 ± 1.20 ^c^	14.68 ± 0.91 ^cd^	2.10 ± 0.20 ^bc^
		CHCl_3_	10.52 ± 0.80 ^f^	27.35 ± 0.15 ^gh^	47.05 ± 0.94	26.45 ± 0.21 ^f^	11.25 ± 0.99 ^de^	2.46 ± 0.14 ^a^
*A. annua* L.	Roots	MeOH	237.03 ± 5.93 ^a^	240.78 ± 1.27 ^a^	438.43 ± 10.59 ^a^	294.52 ± 8.32 ^a^	14.38 ± 0.60 ^c^	2.24 ± 0.08 ^a^
		CHCl_3_	29.98 ± 0.55 ^d^	60.61 ± 0.62 ^d^	87.39 ± 4.62 ^e^	54.97 ± 0.08 ^e^	n.a.	2.37 ± 0.06 ^a^
	Aerial parts	MeOH	102.66 ± 2.15 ^b^	134.36 ± 2.28 ^b^	156.62 ± 4.15	58.67 ± 1.45 ^d^	17.46 ± 3.03 ^bc^	1.55 ± 0.02 ^f^
		CHCl_3_	13.04 ± 0.70 ^f^	32.49 ± 0.34 ^fg^	58.26 ± 1.33	24.79 ± 1.22 ^f^	20.91 ± 1.10 ^ab^	1.85 ± 0.06 ^de^
*A. austriaca* Jacq.	Roots	MeOH	48.99 ± 0.07 ^c^	77.19 ± 0.06 ^c^	168.90 ± 2.70 ^c^	105.77 ± 3.02 ^c^	15.84 ± 0.19 ^b^	1.59 ± 0.05 ^c^
		CHCl_3_	19.68 ± 0.22 ^e^	52.04 ± 2.09 ^e^	82.69 ± 1.76 ^e^	40.68 ± 1.77 ^f^	3.76 ± 0.46 ^g^	1.70 ± 0.05 ^c^
	Aerial parts	MeOH	64.85 ± 0.09 ^d^	75.19 ± 0.42 ^d^	143.59 ± 2.21	59.60 ± 0.61 ^d^	22.16 ± 0.88 ^a^	1.66 ± 0.17 ^ef^
		CHCl_3_	11.05 ± 0.07 ^f^	37.64 ± 0.55 ^f^	52.72 ± 0.55	25.57 ± 2.21 ^f^	12.76 ± 1.19 ^de^	1.56 ± 0.07 ^f^
*A. pontica* L.	Roots	MeOH	179.63 ± 2.60 ^b^	176.12 ± 2.64 ^b^	263.94 ± 1.87 ^b^	165.55 ± 3.83 ^b^	22.93 ± 0.32 ^a^	1.97 ± 0.00 ^b^
		CHCl_3_	31.34 ± 0.41 ^d^	51.69 ± 1.19 ^e^	80.33 ± 1.19 ^e^	45.77 ± 0.74 ^ef^	6.48 ± 0.28 ^ef^	1.53 ± 0.09 ^cd^
	Aerial parts	MeOH	71.65 ± 3.52 ^c^	98.45 ± 3.20 ^c^	290.14 ± 8.95	113.33 ± 1.15 ^b^	9.89 ± 0.99 ^e^	1.55 ± 0.07 ^f^
		CHCl_3_	10.55 ± 1.18 ^f^	25.86 ± 0.50 ^h^	49.99 ± 1.00	22.18 ± 1.39 ^f^	17.84 ± 0.44 ^bc^	1.52 ± 0.10 ^f^
*A. vulgaris* L.	Roots	MeOH	48.83 ± 0.04 ^c^	49.36 ± 0.40 ^ef^	113.97 ± 2.77 ^d^	66.51 ± 2.80 ^d^	5.78 ± 0.13 ^f^	1.23 ± 0.02 ^e^
		CHCl_3_	26.16 ± 0.50 ^d^	63.81 ± 1.19 ^d^	46.26 ± 4.81 ^f^	24.87 ± 2.41 ^g^	n.a.	1.35 ± 0.09 ^de^
	Aerial parts	MeOH	139.56 ± 3.19 ^a^	173.86 ± 3.66 ^a^	498.32 ± 4.02	198.51 ± 5.00 ^a^	12.35 ± 1.15 ^de^	2.33 ± 0.11 ^ab^
		CHCl_3_	33.74 ± 0.49 ^e^	56.54 ± 0.50 ^e^	111.48 ± 2.01	47.73 ± 1.66 ^e^	20.94 ± 1.65 ^ab^	1.89 ± 0.04 ^cd^

Data are presented as mean ± standard deviation (SD) of three determinations; different superscript letters within columns indicate significant differences in the tested extracts for the same parts (*p* < 0.05). ABTS, 2,2′-azino-bis (3-ethylbenzothiazoline) 6-sulfonic acid; CUPRAC, cupric ion reducing antioxidant capacity; DPPH, 1,1-diphenyl-2-picrylhydrazyl; EDTAE, EDTA equivalents; FRAP, ferric ion reducing antioxidant power; MCA, metal chelating activity; n.a., not active; PBD, phosphomolybdenum assay; TE, trolox equivalents.

**Table 4:** Enzyme inhibitory activity of *Artemisia* spp. extracts.

*Artemisia* Species	Part	Extraction Solvent	AChE (mg GALAE/g)	BChE (mg GALAE/g)	Tyrosinase (mg KAE/g)	Amylase (mmol ACAE/g)	Glucosidase (mmol ACAE/g)
*A. absinthium* L.	Roots	MeOH	3.02 ± 0.05 ^a^	1.19 ± 0.11 ^d^	41.20 ± 0.73 ^ab^	0.30 ± 0.01 ^e^	0.88 ± 0.01 ^a^
		CHCl_3_	2.18 ± 0.14 ^b^	2.91 ± 0.21 ^c^	19.44 ± 0.67 ^c^	0.32 ± 0.01 ^e^	0.87 ± 0.01 ^ab^
	Aerial parts	MeOH	2.33 ± 0.02 ^ab^	2.32 ± 0.16 ^bc^	37.09 ± 1.31 ^bcde^	0.40 ± 0.01 ^e^	11.18 ± 0.20 ^a^
		CHCl_3_	2.50 ± 0.00 ^a^	2.67 ± 0.43 ^ab^	35.78 ± 1.53 ^cde^	0.44 ± 0.00 ^cd^	10.85 ± 0.13 ^ab^
*A. annua* L.	Roots	MeOH	2.00 ± 0.06 ^bc^	n.a.	49.42 ± 4.51 ^a^	0.31 ± 0.01 ^e^	0.81 ± 0.01 ^abc^
		CHCl_3_	2.20 ± 0.02 ^b^	4.52 ± 0.18 ^a^	45.74 ± 0.56 ^ab^	0.50 ± 0.02 ^b^	0.87 ± 0.02 ^ab^
	Aerial parts	MeOH	1.97 ± 0.14 ^c^	2.14 ± 0.08 ^bc^	35.71 ± 2.04 ^cde^	0.41 ± 0.02 ^e^	5.93 ± 0.93 ^d^
		CHCl_3_	2.36 ± 0.08 ^ab^	3.11 ± 0.10 ^a^	36.39 ± 2.36 c^de^	0.54 ± 0.01 ^a^	8.84 ± 1.08 ^bc^
*A. austriaca* Jacq.	Roots	MeOH	1.86 ± 0.07 ^cd^	n.a.	47.27 ± 5.68 ^ab^	0.31 ± 0.00 ^e^	0.16 ± 0.03 ^f^
		CHCl_3_	2.16 ± 0.05 ^b^	3.45 ± 0.39 ^b^	13.16 ± 2.73 ^c^	0.57 ± 0.03 ^a^	0.79 ± 0.01 ^bc^
	Aerial parts	MeOH	2.00 ± 0.08 ^c^	1.94 ± 0.55 ^bc^	39.37 ± 0.77 ^abc^	0.42 ± 0.00 ^de^	6.07 ± 0.40 ^d^
		CHCl_3_	2.16 ± 0.02 ^bc^	2.55 ± 0.22 ^abc^	39.37 ± 0.94 ^abcd^	0.54 ± 0.01 ^a^	9.84 ± 0.71 ^abc^
*A. pontica* L.	Roots	MeOH	2.05 ± 0.12 ^cd^	n.a.	44.91 ± 5.05 ^ab^	0.30 ± 0.00 ^e^	0.65 ± 0.05 ^d^
		CHCl_3_	1.82 ± 0.05 ^bc^	0.93 ± 0.06 ^d^	38.30 ± 1.69 ^b^	0.38 ± 0.01 ^d^	0.77 ± 0.01 ^c^
	Aerial parts	MeOH	1.92 ± 0.05 ^c^	1.82 ± 0.05 ^c^	44.64 ± 0.40 ^a^	0.46 ± 0.02 ^c^	6.21 ± 0.79 ^d^
		CHCl_3_	2.15 ± 0.13 ^bc^	2.34 ± 0.02 ^bc^	42.82 ± 2.30 ^ab^	0.50 ± 0.01 ^b^	8.54 ± 0.58 ^c^
*A. vulgaris* L.	Roots	MeOH	1.72 ± 0.08 ^bc^	0.46 ± 0.08 ^e^	41.08 ± 0.68 ^ab^	0.31 ± 0.01 ^e^	0.30 ± 0.07 ^e^
		CHCl_3_	1.98 ± 0.16 ^bc^	0.12 ± 0.01 ^ef^	13.79 ± 2.78 ^c^	0.44 ± 0.02 ^c^	0.87 ± 0.01 ^abc^
	Aerial parts	MeOH	2.04 ± 1.14 ^c^	1.86 ± 0.31 ^c^	31.38 ± 2.74 ^e^	0.40 ± 0.01 ^e^	11.32 ± 0.38 ^a^
		CHCl_3_	2.10 ± 0.12 ^bc^	2.14 ± 0.12 ^bc^	33.23 ± 4.15 ^de^	0.51 ± 0.01 ^ab^	10.01 ± 1.30 ^abc^

Data are presented as mean ± standard deviation (SD) of three determinations; different superscript letters within columns indicate significant differences in the tested extracts for the same parts (*p* < 0.05). ACAE, acarbose equivalents; AChE, acetylcholinesterase; BChE, butyrylcholinesterase; GALAE, galanthamine equivalents; KAE, kojic acid equivalents; n.a., not active.

**Table 5:** Anti-*Mycobacterium tuberculosis* H37Ra activity of *Artemisia* spp. extracts.

*Artemisia* Species	Part	Extraction Solvent	MIC (mg/L)
*A. absinthium* L.	Roots	MeOH	>256
		CHCl_3_	256
	Aerial parts	MeOH	256
		CHCl_3_	128
*A. annua* L.	Roots	MeOH	256
		CHCl_3_	128
	Aerial parts	MeOH	256
		CHCl_3_	128
*A. austriaca* Jacq.	Roots	MeOH	>256
		CHCl_3_	256
	Aerial parts	MeOH	128
		CHCl_3_	64
*A. pontica* L.	Roots	MeOH	256
		CHCl_3_	128
	Aerial parts	MeOH	256
		CHCl_3_	256
*A. vulgaris* L.	Roots	MeOH	>256
		CHCl_3_	128
	Aerial parts	MeOH	256
		CHCl_3_	128
Etambutol	–	–	2
Streptomycin	–	–	0.5
Rifampicin	–	–	0.002

## Data Availability

Not applicable.

## Supplementary Materials

The following supporting information can be downloaded at: https://www.mdpi.com/article/10.3390/antiox11051017/s1, Table S1. Full spectro-chromatographic data of compounds identified in the *Artemisia* root and aerial parts extracts by LC-HRMS/MS.
